# Weight assessment and the provision of weight management advice in primary care: a cross-sectional survey of self-reported practice among general practitioners and practice nurses in the United Kingdom

**DOI:** 10.1186/s12875-020-01184-z

**Published:** 2020-06-19

**Authors:** Nathan Critchlow, Gillian Rosenberg, Harriet Rumgay, Robert Petty, Jyotsna Vohra

**Affiliations:** 1 Cancer Policy Research Centre, Cancer Research UK, 2 Redman Place, London, E20 1JQ UK; 2 Institute for Social Marketing and Health, Faculty of Health Sciences and Sport, University of Stirling, Stirling, FK9 4LA UK; 3 Cancer Intelligence, Cancer Research UK, 2 Redman Place, London, E20 1JQ UK

**Keywords:** Obesity, Weight management, Primary care, Survey, Weight assessment, Overweight, General practitioners, Nurses, Obesity policy

## Abstract

**Background:**

Although primary care settings provide a large-scale and high-reach opportunity for weight management and obesity prevention, the proportion of adults in the United Kingdom (UK) who report receiving weight management advice is limited. This study examines the self-reported frequency of assessing weight and providing weight management advice by General Practitioners (GPs) and Practice Nurses (PNs) working in primary care in the UK, and differences by practitioner characteristics.

**Methods:**

Cross-sectional survey with GPs and PNs in the UK (*n* = 2020), conducted January–March 2017. A mock consultation exercise assessed what factors led to calculating a patient’s Body Mass Index (BMI) and whether weight management advice was given after determining the patient had an obese BMI. For all patients, practitioners were asked how often they calculated BMI, how often they gave weight management advice to patients with an obese BMI, and how often they utilised different advice or referral options (each: *Always/Often* vs. *Less often/Never*). Binary logistic regressions examined whether frequency of assessing weight and providing advice was associated with practitioner characteristics.

**Results:**

In the mock consultation, physical cues (40%) were most likely to prompt calculation of BMI, and half of practitioners (56%) provided weight management advice after determining the patient had an obese BMI, with GPs less likely to do so than PNs (*Odds Ratio [OR]* = 0.59, 95% CI: 0.47–0.75). Half of practitioners (58%) said they calculated the BMI of all patients Always/Often, with GPs less likely to do so than PNs (*OR* = 0.27, 95% CI: 0.21–0.34). Three quarters (78%) said they provided weight management advice to patients with an obese BMI Always/Often, with GPs less likely to do so than PNs (*OR* = 0.63, 95% CI: 0.47–0.85). Weight management advice was provided more frequently than referrals, particularly suggesting increased physical activity (93%) and diet modification (89%).

**Conclusions:**

Consistent with previous research, the findings suggest that opportunities to provide weight management advice in primary care, including to patients with an obese BMI, are potentially missed. Future research should test alternative mechanisms to increase weight assessment and advice provision, examine the effectiveness of advice frequently given, and seek solutions to reported barriers for providing weight management advice.

## Background

In England, over a third (35.6%) of adults have an overweight Body Mass Index (BMI) and almost another third (28.7%) have an obese BMI [1]. In 2017/2018, there were 10,660 hospital admissions directly linked to obesity in England and nearly three quarters of a million admissions where obesity was a factor [2]. This has economic implications, as the National Health Service (NHS) spends an estimated £6.1 billion on overweight and obesity-related ill-health each year, and the wider costs to society are estimated at £27 billion [3]. Obesity has also personal health implications, for example it is linked to multiples types of cancer [4] and over 20,000 cancer cases each year in the United Kingdom (UK) [5].

As most of the population in the UK are registered with a General Practitioner (GP) practice [6], primary care settings provide a large-scale and high-reach opportunity for obesity intervention and prevention. Research suggests that well-planned and adequately resourced brief interventions delivered in primary care can be effective in stimulating weight loss, particularly when discussions generate referrals to specialist weight management services [7–15]. In the UK, primary care practitioners are therefore encouraged to instigate brief conversations to help identify at-risk patients (including calculating BMI as part of their assessment), explain behaviour modifications that would lead to weight reductions, and make referrals to specialist weight management services if required [16]. Similar brief interventions are also advised for smoking cessation and higher-risk alcohol consumption [17–19].

Patients believe that primary care practitioners play an important role in weight management are expectant they will discuss the issue if required [20, 21], while primary care practitioners similarly acknowledge they play a key role in obesity prevention [22, 23]. A nationally representative survey, however, suggests that only around a fifth of adults in the UK have received advice about weight management from a healthcare practitioner, including less than half of those who had an obese BMI [24]. This is despite the same study finding that almost all adults had seen a healthcare practitioner in the past year. These trends are corroborated in wider examinations of patient records, self-reported frequency of assessing weight and providing referrals among primary care practitioners, and observations of primary care consultations [11, 25–32]. Moreover, even when weight is assessed and a referral to a specialist weight management service is made, only around a third of patients reportedly attend the referral at all and fewer complete the referral in full [33]. Combined, research therefore suggests that opportunities to provide weight management advice to patients in primary care settings, including those at risk, are potentially being missed.

Examining self-reported provision of weight management advice among primary care practitioners provides important insight into perceived normative practice. Although there is existing research concerning this topic in the UK, most studies are based on smaller qualitative samples, only focus on specific parts of the UK, or only focus on certain practitioner groups. There is a need for larger studies which examine self-reported practice across the UK and assess to what extent, if at all, there is variation among primary care practitioners (e.g. by experience, role, or country). In response, we examine self-reported provision of weight management advice among a large sample of GPs and Practice Nurses (PNs) from across the UK. We examine what factors prompt weight assessment in primary care, how often they report providing weight management advice, what advice or referrals are provided frequently, and how provision of weight management advice varies by practitioner characteristics.

## Methods

### Design and sample

An online cross-sectional survey was conducted with primary care health practitioners in the UK (*n* = 2020). Data were collected between January and March 2017. The survey only included PNs and GPs; other health professionals, such as physiotherapists and dentists, were excluded through screening questions. ResearchNow (now called Dynata), the market research company who conducted the survey, recruited a convenience sample through their existing panel of health professionals. A survey weight was provided to enable descriptive data to be representative of participant country within the UK (England, Scotland, Wales, and Northern Ireland). The survey included sections about the delivery of brief advice related to weight, smoking [34], and alcohol consumption in primary care. This study only focuses on the data concerning weight management. The survey design and content was developed and piloted using a health professional panel made up of eight Cancer Research UK health facilitators who work with primary care practitioners [34].

### Measures

#### Primary care practitioner characteristics

Practitioners were asked to self-report their gender, age (coded: 18–39 years, 40–59 years, > 60 years), healthcare professional role (GP or PN), years qualified (coded: 0–5 years, 6–10 years, 11–15 years, 16–20 years, > 20 years), typical days worked in general practice per week (coded: 1–2 days, 3–4 days, 5–6 days), approximate list size of practice where they worked (coded: < 2000 patients; 2000-5000; 5000-10,000; 10,000-20,000; > 20,000), and in which Clinical Commissioning Group (CCG [35]) or Health Board [36, 37] their practice was located. The latter variable was used to assign country (coded: England, Scotland, Wales, and Northern Ireland).

#### Providing weight management advice in a mock consultation

To prompt whether practitioners would provide weight management advice during a typical patient consultation, participants were presented with a brief mock scenario in the form of a short written vignette. Use of vignettes to prompt practitioner reactions have been used elsewhere in primary care research [38–40]. Vignettes were tailored for GPs and PNs to reflect the different types of patients that they would typically consult in primary care. For GPs, the brief vignette read ‘*Maya is a 52 year old female who presents with a persistent cough*’. For PNs, the brief vignette read ‘*Maya is a 52 year old female who presents with a burn that requires wound management’*. Consultations deliberately did not present issues directly related to weight to avoid response bias. Three questions were asked in response to this prompt.

First, practitioners were asked what factors were most likely to result in them calculating the patient’s BMI. They were presented with seven response options: [1] previous weight-related health condition [2]; weight-associated symptoms [3]; physical cues, e.g. size/body shape [4]; computer prompts [5]; incentives payments [6]; known increased or high BMI from medical records [7]; National Institute for Health and Care Excellence [NICE] weight loss guidelines; and [8] other reasons, with free text box provided. Practitioners could only select one option.

Second, practitioners were then prompted with the statement ‘*Maya has a BMI of 32 and asks whether her weight is a problem. How would you respond?’* A free text box was provided for answers. Free text responses were binary coded based on whether the practitioner’s answer suggested they would provide weight management advice, take action (e.g. make a referral), or start a conversation about weight management (Yes/No). Answers coded as ‘No’ included simply informing the patient they were overweight, stating the associated health risks of being overweight, or other unclear or administrative actions (e.g. checking medical records). One member of the research team manually coded all response options, and a second researcher subsequently checked the coding. Any discrepancies or uncertainty was discussed and agreed between the two researchers.

Third, practitioners were asked the minimum BMI which would lead to them starting a conversation with the patient about weight management. Answers were provided on a scale from 20.0 to 40.0 BMI. Answers were coded as being within either the healthy ([Min]20.0–24.9), overweight (25.0–29.9), or obese (> 30.0) BMI range for adults.

#### Frequency of calculating BMI for all patients

Practitioners were asked to think about all the patients they had seen at their practice in the last year and asked ‘*How often did you calculate a patient’s BMI*?’ Answers were provided on a five-point scale (*1 = Always–5 = Never*) and binary coded (*1 = Always/Often, 0 = Less often/Never*).

#### Provision of weight management advice to patients with an obese BMI

Practitioners were asked ‘*Thinking now about all the patients that you saw in the last year who are obese (with a recorded BMI of 30-35), how often did you provide weight management advice?’* Answers were provided on a five-point scale (*1 = Always–5 = Never*) and binary coded (*1 = Always/Often, 0 = Less often/Never*).

#### Advice and referrals in weight management

Practitioners were promoted with ‘*For all patients in the last year that you gave advice about weight management, how often did you … ’* and then provided with 14 outcomes, including 5 examples of advice (e.g. increase physical activity or diet modification) and eight examples of referrals (e.g. make a referral to a dietician/healthy eating course within the practice) (see Table 5 for full details). Answers to each option were provided on a five-point scale (*1 = Always–5 = Never*) which was binary coded (*1 = Always/Often, 0 = Less often/Never*).

### Analysis

All data were analysed using SPSS version 24 (SPSS Inc., Chicago). Weighted and unweighted frequencies were calculated for practitioner characteristics. Weighted frequencies were also calculated for each of the main study variables, for example what factor was most likely to stimulate calculation of BMI in the mock consultation and whether BMI was calculated for all patients Always/Often (vs. Less often/Never). For each variable, Pearson Chi-square tests examined differences by gender, age, days spent in practice in a typical week, years qualified, list size of the practice, and country.

Binary logistic regression models examined to what extent, if at all, weight assessment and provision of weight management advice was associated with practitioner characteristics. Four models were computed: [1] whether weight management advice was given in the mock consultation (*Yes/No*) [2]; the minimum BMI that would instigate a conversation about weight management in the mock consultation (*obese BMI* vs. *overweight BMI*) [3]; how often BMI was calculated for all patients (*Always/Often* vs. *Less often/Never*); and [4] how often weight management advice was given to those with a BMI in the obese range (*Always/Often* vs. *Less often/Never*). Gender, age group, typical days worked in practice, practitioner role, years qualified, list size of practice, and country of residence were included as covariates. Reference categories for categorical variables with < 2 levels (e.g. practitioner role) are reported in the results. For country, the simple = contrast function compared each of Scotland, Wales, and Northern to England. For days worked in practice, years qualified and list size, both which had > 3 levels and were ordinal data, the contrast = difference function enabled comparison of each increasing category relative to the combined preceding levels. For example, the first comparison for years qualified was 6–10 years versus 0–5 years, whereas the next was 11–15 years versus less often (i.e. combined 0–5 and 6–10 years categories). The binary regressions were conducted on unweighted data, as the variable used to construct weights (country) was included as a covariate.

## Results

### Sample characteristics

In the weighted sample, there was an even proportion of GPs and PNs (Table 1). Most GPs were male (62%), aged 40–59 years old (55%), based in England (84%), and typically worked in practice at least 3–4 days per week. More than half of GPs had been qualified for > 16 years and worked in a practice with a list size of at least 5000 patients. Almost all PNs were female (95%). The majority of PNs were aged 40–59 years old (65%), based in England (84%), typically worked in practice 3–4 days per week (61%), had been qualified > 20 years (56%), and worked in practice with a list size of at least 5000 patients. Overall, practitioners came from 234 CCGs or Health Boards across the UK, suggesting good geographical coverage.

**Table 1:** Weighted sample characteristics, by practitioner role

Characteristics	Overall (*n* = 2020)		PNs (*n =* 1014)		GPs (*n =* 1006)	
	%	***n***	%	***n***	%	***n***
**Gender**						
Female	66	1339	95	960	38	378
Male	34	681	5	54	62	627
**Age**						
18–39 years	34	680	28	280	40	400
40–59 years	60	1216	65	662	55	554
60+ years	6	124	7	72	5	52
**Country**						
England	84	1693	84	852	84	841
Scotland	9	172	8	82	9	90
Wales	5	99	5	51	5	48
Northern Ireland	3	57	3	30	3	27
**Typical days in general practice**						
1–2 days per week	7	149	9	91	6	58
3–4 days per week	59	1184	61	619	56	565
5–6 days per week	34	687	30	304	38	383
**Years qualified**						
0–5 years	8	171	8	82	9	89
6–10 years	15	308	12	120	19	188
11–15 years	16	331	13	133	20	198
16–20 years	15	306	11	115	19	191
More than 20 years	45	905	56	565	34	340
**List size at practice**						
< 2000	4	76	6	58	2	18
2000–5000	15	314	15	151	16	163
5000–10,000	37	739	34	348	39	391
10,000–20,000	36	717	33	336	38	381
> 20,000	5	107	6	64	4	43
Unsure	3	67	6	58	1	9

Notes

Data are weighted

^1^ = Practice Nurse.

^2^ = General Practitioner.

### Mock consultation: what factors most likely lead to calculating BMI?

In the mock consultation, physical cues were identified by most practitioners as the factor most likely to prompt them to calculate the patient’s BMI (40% of practitioners). This was followed by weight-related symptoms (29%) and computer prompts (12%) (Table 2). Compared to PNs, Chi-square tests found that GPs were more likely to calculate BMI due to physical cues (*p* = 0.036, *ϕ* (Phi) = 0.05), weight-related symptoms (*p* < 0.001, *ϕ* = 0.22), and incentives (*p* < 0.001, *ϕ* = 0.08). Compared to GPs, PNs were more likely to calculate BMI due to computer prompts (*p* < 0.001, *ϕ* = − 0.20), previous weight-related health conditions (*p* = 0.003, *ϕ* = − 0.07), knowing about increasing BMI from medical records (*p* < 0.001, *ϕ* = − 0.15), and for other reasons (*p* < 0.001, *ϕ* = − 0.09).

**Table 2:** Factors most likely to prompt calculation of Body Mass Index (BMI) in mock consultation, by practitioner role

Prompt	Overall		PN^**1**^		GPs^**2**^		Chi-Square	
	%	*n*	%	*n*	%	*n*	χ^2^	*p*
Physical cues (e.g. body size)	40	813	38	385	43	428	4.40	0.036
Weight-related symptoms	29	585	19	193	39	392	97.83	< 0.001
Computer prompt	12	238	18	183	6	55	76.89	< 0.001
Previous weight-related health condition	7	136	8	85	5	51	8.76	0.003
Known increasing or high BMI^3^ from medical records	6	128	10	102	3	26	47.54	< 0.001
Incentives payments	2	42	1	9	3	33	14.25	< 0.001
NICE weight loss guidelines	2	36	2	23	1	13	2.74	n.s.
Other	2	41	3	34	1	7	17.90	< 0.001

Notes

Base = All participants; Data and analyses are weighted

Response options are sorted by proportion (%) reported, based on overall sample

^1^ = Practice Nurse.

^2^ = General Practitioner.

^3^ = Body Mass Index (*BMI*), *n.s* not significant (*p* > 0.05)

### Mock consultation: responding to patient BMI of 32

When prompted with the statement ‘*Maya has a BMI of 32 and asks whether her weight is a problem. How would you respond?’* around half of practitioners (56%) provided a free text answer coded as giving advice about weight management, taking action, or starting a conversation about weight management. A binary logistic regression found that responding with advice, action, or starting a conversation was lower in males compared to females (*Adjusted Odds Ratio [AOR]* = 0.70, *p* = 0.004) and among GPs compared to PNs (*AOR* = 0.59, *p* < 0.001) (Table 3). Responding with advice, action or starting a conversation was greater for practitioners in Wales (*AOR =* 1.74, *p* = 0.016) and Scotland (*AOR* = 1.40, *p* = 0.043), compared to England.

**Table 3:** Binary logistic regressions exploring the association between practitioner characteristics and (1) whether weight management advice was provided in mock consultation and (2) whether conversations would be initiated at overweight or obese Body Mass Index (BMI)

Variables/Reference categories	Provide advice to mock consultation BMI of 32?^**1,3,5**^			What BMI trigger weight management conversation? ^**2,4,6**^		
	***AOR***	95% CI	***p***	***AOR***	95% CI	***p***
**Gender**						
Female	REF	–	–	REF	–	–
Male	0.70	0.55–0.89	0.004	0.59	0.46–0.76	< 0.001
**Age**						
18–39 years old	REF	–	n.s.	REF	–	n.s.
40–59 years old (vs younger)	1.08	0.79–1.49	n.s.	1.20	0.86–1.67	n.s.
> 60 years old (vs younger)	1.00	0.65–1.55	n.s.	0.77	0.49–1.21	n.s.
**Country**						
England	REF	–	0.024	REF		0.052
Scotland (vs. Eng)	1.40	1.01–1.93	0.043	0.79	0.57–1.09	n.s.
Wales (vs. Eng)	1.74	1.11–2.73	0.016	0.63	0.41–0.98	0.041
Northern Ireland (vs. Eng)	0.95	0.57–1.58	n.s.	0.65	0.38–1.13	n.s.
**Days in practice in week**						
1–2 days	REF	–	n.s.	REF	–	n.s.
3–4 days (vs. less often)	1.01	0.69–1.47	n.s.	0.81	0.53–1.21	n.s.
5–6 days (vs. less often)	0.81	0.63–1.03	n.s.	1.11	0.85–1.45	n.s.
**Health professional role**						
Practice nurse	REF	–	–	REF	–	–
General practitioner	0.59	0.47–0.75	< 0.001	0.59	0.47–0.76	< 0.001
**Years qualified**						
0–5 years	REF	–	n.s.	REF	–	n.s.
6–10 years (vs. more recent)	0.91	0.61–1.35	n.s.	0.90	0.60–1.37	n.s.
11–15 years (vs. more recent)	0.95	0.70–1.30	n.s.	0.80	0.57–1.10	n.s.
16–20 years (vs. more recent)	0.92	0.65–1.31	n.s.	0.86	0.60–1.23	n.s.
> 20 years (vs. more recent)	1.02	0.77–1.35	n.s.	0.89	0.66–1.20	n.s.
**List size**						
< 2000 patients.	REF	–	n.s.	REF	–	n.s.
2000–5000 patients (vs less)	1.30	0.77–2.18	n.s.	1.20	0.67–2.11	n.s.
5000–10,000 patients (vs less)	1.12	0.83–1.51	n.s.	1.09	0.79–1.52	n.s.
10,000–20,000 patients (vs less)	1.21	0.95–1.53	n.s.	0.90	0.70–1.16	n.s.
> 20,000 patients (vs less)	0.77	0.51–1.17	n.s.	1.14	0.73–1.79	n.s.

Notes

Base = All participants; *AOR* = Adj. Odds Ratio; 95% CI = 95% Confidence Interval, *n.s*. non-significant (*p* > 0.05)

Data are not weighted, as country included as a covariate

^1^ DV = Provided advice, conversation or referral in response to prompt (Yes = 1; No = 0).

^2^ DV = Min BMI that would trigger weight conversation (Overweight = 1; Obese = 0).

^3^ Test of coefficients, χ^2^(17) = 95.21, *p* < 0.001; Hosmer & Lemeshow, χ^2^(8) = 12.43, *p* = 0.13, Nagelkerke *R*^*2*^ = 0.06.

^**4**^ Test of coefficients, χ^2^(17) = 108.18,*p* < 0.001; Hosmer & Lemeshow, χ^2^(7) = 2.86, *p* = 0.90, Nagelkerke *R*^*2*^ = 0.08.

^5^ Cases excluded due to missing data on one or more variable (*n* = 68).

^6^ Cases excluded due to missing data on one or more variable (*n* = 193).

### Mock consultation: minimum BMI that would initiate conversation about weight

After accounting for missing data (*n* = 77, weighted), approximately half of practitioners (52%) said the minimum BMI that would trigger a conversation about weight management in the mock consultation would be in the overweight range (BMI: 25.0–29.9), while almost half (45%) said within the obese range (BMI > 30.0). Only a minority (3%) said they would have a conversation when BMI was in the healthy range (BMI 20.0–24.9). A binary logistic regression found that initiating a conversation about weight when the patient was in the overweight BMI range (versus obese) was lower in males compared to females (*AOR* = 0.59, *p* < 0.001), in GPs compared to PNs (*AOR* = 0.59, *p* < 0.001), and for practitioners in Wales compared to England (*AOR* = 0.63, *p* = 0.041) (Table 3).

### Frequency of calculating BMI for all patients

When asked to consider all patients they had seen at their practice in the past year, almost three-fifths of practitioners (58%) said that they had calculated BMI always or often. A binary logistic regression indicated that assessing BMI Always/Often (vs. Less often/Never) was lower in males compared to females (*AOR* = 0.71, *p* = 0.006), in those from Scotland compared to England (*AOR* = 0.69, *p* = 0.030), and in GPs compared to PNs (*AOR =* 0.27, *p* < 0.001) (Table 4). There was also a main association of typical days worked in practice (*p* < 0.001), with those who worked more days per week more likely to measure BMI Always/Often.

**Table 4:** Binary logistic regressions exploring associations between practitioner characteristics and (1) frequency of calculating patient BMI (all patients) and (2) frequency of providing weight management advice to obese patients

Variables/Reference categories	How often calculate patient BMI?^**1,3,5**^			How often provide weight management advice to obese patients? ^**2,4,6**^		
	***AOR***	95% CI	***p***	***AOR***	95% CI	***p***
**Gender**						
Female	REF	–	–	REF	–	–
Male	0.71	0.55–0.91	0.006	0.60	0.45–0.80	< 0.001
**Age**						
18–39 years old	REF	–	n.s.	REF	–	n.s.
40–59 years old (vs younger)	1.06	0.76–1.49	n.s.	1.09	0.74–1.59	n.s.
> 60 years old (vs younger)	1.04	0.65–1.64	n.s.	0.92	0.54–1.54	n.s.
**Country**						
England	REF	–	n.s.	REF		0.006
Scotland (vs. Eng)	0.69	0.50–0.97	0.030	0.76	0.53–1.10	n.s.
Wales (vs. Eng)	1.17	0.74–1.86	n.s.	0.96	0.57–1.63	n.s.
Northern Ireland (vs. Eng)	1.00	0.56–1.66	n.s.	0.40	0.24–0.68	0.001
**Days in practice in week**						
1–2 days	REF	–	< 0.001	REF	–	0.010
3–4 days (vs. less often)	1.64	1.12–2.44	0.013	1.28	0.83–1.98	n.s.
5–6 days (vs. less often)	1.70	1.30–2.21	< 0.001	1.57	1.16–2.12	0.003
**Health professional role**						
Practice Nurse	REF	–	–	REF	–	–
General practitioner	0.27	0.21–0.34	< 0.001	0.63	0.47–0.85	0.002
**Years qualified**						
0–5 years	REF	–	n.s.	REF	–	n.s.
6–10 years (vs. more recent)	1.27	0.84–1.92	n.s.	0.71	0.44–1.16	n.s.
11–15 years (vs. more recent)	1.13	0.82–1.57	n.s.	1.06	0.73–1.55	n.s.
16–20 years (vs. more recent)	1.26	0.87–1.82	n.s.	0.84	0.55–1.27	n.s.
> 20 years (vs. more recent)	1.13	0.83–1.52	n.s.	1.07	0.75–1.53	n.s.
**List size**						
< 2000 patients.	REF	–	n.s.	REF	–	n.s.
2000–5000 patients (vs less)	1.62	0.94–2.79	n.s.	1.85	1.00–3.41	n.s.
5000–10,000 patients (vs less)	1.22	0.89–1.68	n.s.	1.22	0.85–1.75	n.s.
10,000–20,000 patients (vs less)	1.11	0.86–1.42	n.s.	1.04	0.78–1.38	n.s.
> 20,000 patients (vs less)	1.08	0.69–1.69	n.s.	1.21	0.71–2.07	n.s.

Notes

Base = All participants; *AOR* = Adj. Odds Ratio; 95% CI = 95% Confidence Interval; n.s. = non-significant (*p* > 0.05)

Data are not weighted, as country included as covariate

^1^ DV = How often calculate BMI of patient (Always/often = 1; Less often = 0).

^2^ DV = How often provide weight management advice to obese patients (Always/often = 1; Less often = 0).

Model summaries for final block:

^3^ Test of coefficients, χ^2^(17) = 270.13,*p* < 0.001; Hosmer & Lemeshow, χ^2^(8) = 4.29, *p* = 0.83, Nagelkerke *R*^*2*^ = 0.17.

^**4**^ Test of coefficients, χ^2^(17) = 79.54,*p* < 0.001; Hosmer & Lemeshow, χ^2^(8) = 5.78, *p* = 0.67, Nagelkerke *R*^*2*^ = 0.06.

^5^ Cases excluded due to missing data on one or more variable (*n* = 68).

^6^ Cases excluded due to missing data on one or more variable (*n* = 68).

### Providing weight management advice to all patients with obese BMI

Practitioners were asked to consider how often, in the past year, they had given weight management advice to patients with an obese BMI. Over three quarters of practitioners (78%) said they had provided advice to such patients Always/Often. A binary logistic regression found that providing advice Always/Often was lower in males compared to females (*AOR* = 0.60, *p <* 0.001), those in Northern Ireland compared to England (*AOR* = 0.40, *p* = 0.001), and in GPs compared to PNs (*AOR* = 0.63, *p* = 0.002) (Table 4). There was also a main association of typical days worked in practice (*p* = 0.01), with those who typically worked 5–6 days per week more likely to provide advice Always/Often than those who worked less frequently (*AOR =* 1.57, *p* = 0.003).

### Weight management advice given to patients with an obese BMI

Practitioners were asked to consider how often, in the past year, they had provided different weight management advice to patients with an obese BMI (each coded: *Always/Often* vs. *Less often/Never*). The majority of practitioners said they would suggest increased physical activity (93%) or diet modification (89%) Always/Often, while around half (49%) said they would suggest arranging a follow-up appointment to discuss or provide information leaflets (46%) (Table 5). A third (32%) said they would suggest keeping a food diary always or often.

**Table 5:** What advice or referrals did GPs and PNs provide always or often (vs less often) in weight management advice in the past year?

Advice or referral activities	Overall		PN^**1**^		GPs^**2**^		Chi-Square	
	%	*n*	%	*n*	%	*n*	χ^2^	*p*
***Advice***								
Increase physical activity	93	1879	91	926	95	953	9.48	0.002
Diet modification	89	1801	88	893	90	908	2.70	n.s.
Arrange follow-up appointment to discuss	49	979	60	609	37	370	109.08	< 0.001
Provide information leaflet	46	939	66	670	27	269	313.23	< 0.001
Keep food diary	32	649	41	415	23	234	72.04	< 0.001
***Referral***								
Refer to external exercise referral scheme	26	514	27	274	24	240	2.67	n.s.
Refer to NHS weight management programme external to practice	24	475	26	262	21	213	5.99	0.014
Refer to external dietician/healthy eating course	23	458	25	253	20	205	5.96	0.015
Refer to internal dietician/healthy eating course	20	405	23	232	17	173	10.10	0.001
Refer to NHS weight management programme in practice	17	336	22	225	11	111	45.05	< 0.001
Refer to commercial weight loss programme	14	286	17	173	11	113	14.12	< 0.001
Refer to internal exercise referral scheme	13	270	17	174	10	96	25.22	< 0.001
Provide prescription (e.g. Orlistat / Xenical / Alli)	7	146	5	50	10	96	16.02	< 0.001

Notes

Base = All participants; Data and analyses are weighted by country

All data coded Always/Often vs. Less often (e.g. Sometimes, Occasionally, Never); % shown are those providing Always/often

^1^ = Practice Nurse.

^2^ = General Practitioner.

*n.s.* non-significant (*p* > 0.05)

Bivariate Pearson Chi-square tests found that PNs were more likely than GPs to Always/Often suggest arranging a follow-up appointment (*p* < 0.001, *ϕ* = − 0.23), provide information leaflets (*p* < 0.001, *ϕ* = − 0.39) or suggest keeping a food diary (*p* < 0.001, *ϕ* = − 0.19) (Table 5). Compared to PNs, Chi-square tests found that GPs were more likely to suggest increasing physical activity (*p* = 0.002, *ϕ* = 0.07). There was no difference between GPs and PNs for suggesting diet modification.

### Weight management referrals given to patients with an obese BMI

Practitioners were asked to consider how often, in the past year, they had given different referral options to patients with an obese BMI (each coded: *Always/Often* vs. *Less often/Never*). Approximately a quarter said they would always or often refer patients to an external exercise referral scheme (26%), an NHS weight management programme external to the practice (24%), or a dietician or healthy eating course external to the practice (23%). Around a fifth said that they would refer patients to a dietician or healthy eating course internal to the practice (20%) or to an NHS weight management programme in the practice (17%) (Table 5). At least one-in-10 practitioners said that they would refer patients to a commercial weight loss programme (14%) or an exercise referral scheme internal to the practice (13%). Less than one-in-10 said that they would provide a prescription (7%).

Compared to GPs, Chi-square tests found that PNs were more likely to refer patients Always/Often for six of the eight outcomes (Table 5). The only exceptions were providing prescriptions, an outcome more likely among GPs (*p <* 0.001, *ϕ* = 0.09), and referral to exercise referral scheme external to the practice, where there was no difference between GPs and PNs.

## Discussion

By exploring self-reported practice among a large sample GPs and PNs, and examining differences by practitioner characteristics, we provide further understanding about weight assessment and the provision of weight management advice in UK primary care settings. Concerning assessment, around half of practitioners reported that they frequently calculated BMI in all patients, however no single factor prompted the majority to do this in the mock consultation. Concerning advice, only around half of practitioners provided weight management advice in the mock consultation after determining that the patient had an obese BMI. This estimate is lower than was self-reported for all patients seen in the last year who had an obese BMI, in which around three quarters said they frequently provided weight management advice, albeit this may be related to the explicitness of the latter question. Concerning action, practitioners reportedly provided weight management advice more frequently than referrals, particularly suggestions to increase physical activity and modify diet.

Clinical guidelines [41], performance indicators [42], and recommendations of best practice [16, 43] suggest that practitioners working in primary care in the UK should regularly assess patient BMI and, where required, instigate discussions about weight management and make appropriate referrals to weight management services. In this study, however, only around half of practitioners provided weight management advice in the mock consultation, only around half said they frequently assessed BMI among all patients, and only three quarters said they frequently provided weight management advice to patients with an obese BMI. These findings are therefore consistent with infrequent reports of providing or receiving advice in patient research, self-report research with primary care practitioners, observations of consultations, and examinations of patient records [24–32].

Research with primary care practitioners has suggested several barriers to routinely discussing weight with patients, which may partly explain these findings. Examples include lack of time or competing priorities, knowledge of obesity guidelines or relevant training, concerns of sensitivity or negative consequences (e.g. upsetting patients), and scepticism about the efficacy of advice [22, 23, 44–51]. Similar themes have also been reported in patient-focused research [20, 21, 31]. Some of these barriers are also reportedly aggravated among GPs, in particular concerns about moving the consultation away from the patient’s agenda, and harming their doctor-patient relationship [45]. This may help to explain why PNs were more likely to report frequently assessing BMI and more likely to provide weight management. This is in addition to wider suggestions that believing weight to be part of their chronic disease and health promotion remit, confidence in their ability to build patient rapport, and being encouraged to attend training and make time for weight management may also facilitate more frequent weight management practice among PNs [22, 45, 52]. That almost all PNs were female also explains why gender was also associated with greater likelihood of, or more frequent, weight management practice. Wider factors which may also influence provision of weight assessment and management include adequate local-level funding, practitioner knowledge of available and effective referral opportunities, tensions around whether obesity is a medical or social problem, and effective communication between primary care and weight management services [14, 15, 53].

The findings highlight four considerations for future practice and research. First, no single factor was identified by the majority of practitioners as prompting them to calculate BMI. Future research should therefore explore whether increased emphasis on any of the lesser reported prompts could increase assessment and advice provision, with previous research supporting the potential efficacy of computer prompts [54, 55], financial incentives [56, 57], or multicomponent interventions [14]. It is also important that any alternatives are implemented in a manner that reduces, or avoids exacerbating, concerns among patients that they are being stigmatised [31] or existing barriers reported among practitioners. Put simply, continued examination and evaluation of what works, and why, for practitioners and patients is essential. Second, only around half of practitioners said the minimum BMI that would trigger a conversation about weight management was in the overweight range. Given the potential effectiveness of brief primary care advice [7–14], research should explore whether increasing the proportion of earlier interventions in the overweight range reduces escalation to obesity and some of the perceived barriers (e.g. concerns about offending patients). Third, the findings highlight what forms of advice or referrals were used most frequently. Further examination is needed to determine the efficacy and cost-effectiveness of these actions, how they compare to alternative options given less frequently, and what barriers exist to providing referrals versus advice (e.g. availability of local services or patient receptiveness). For example, research with patients has suggested that perceived simplistic advice around consumption and exercise, the two forms of advice provided most frequently in this study, may have limited resonance with some patients [31]. Finally, it is possible that differences in weight management practice between GPs and PNs, and the barriers reported elsewhere [44–51], may relate to inherent differences in their professional roles, responsibilities, and nature of patient contact. It may, therefore, be beneficial for research to further examine PN and GPs separately to examine best practice, barriers, and opportunities for focussed interventions among each group.

There are limitations. The data are cross-sectional and only show associations between provision of weight management and practitioner characteristics, they cannot determine underlying causal influence. Practitioners were drawn from a convenience sample recruited by a market research company, and findings may not be representative of all GPs and PNs working in primary care. The data only come from a single time point in early 2017, and may not be representative of other times of the year or changes in practice since data collection. The data were self-reported, and therefore provision of weight management advice may be underestimated due to errors in recall or outcomes not included as response options. The possibility of response bias cannot be discounted, particularly when the question implied what the logical practice was (e.g. giving weight management advice to patients with an obese BMI).

A further important limitation is that the mock consultation only provided a limited example of patient contact, and it cannot be known if practitioners would have reacted different in-person (e.g. using non-verbal cues) or if the original health concern was different or had links to weight. We also acknowledge that providing written descriptions of how they would respond in the mock consultation does not reflect the discursive and verbal nature of how this advice is actually given. The mock consultation also cannot fully reflect or accurately recreate important contextual aspects of consultations, namely existing patient relationships, prior knowledge of the patient’s weight behaviour or health conditions, or time limitations. Introducing this information into the vignette could have biased responses (i.e. if stating presence or absence of health conditions), reduced applicableness to some practitioners (e.g. newly qualified practitioners may not have longstanding patient relationships), or reduced generalisability to some contexts (e.g. a prior relationship is a not prerequisite for all primary care consultation scenarios). Other methods, such as observations during practice or qualitative interviews, are better equipped to examine the relative influence of contextual factors.

## Conclusion

By exploring the self-reported behaviour of a large sample GPs and PNs, and differences by practitioner characteristics, we provide further understanding about weight assessment and the provision of weight management advice in UK primary care settings. For assessment, only around half of practitioners reported frequently calculating BMI, and there was no single factor likely to prompt the majority of practitioners to do this. Only around half of practitioners provided weight management advice in the mock consultation, despite being informed that the patient had an obese BMI, and only three-quarters said they did this frequently for all patients with an obese BMI. Concerning action, practitioners provided weight management advice to patients with obese BMI more frequently than referrals. The findings highlight several potential next steps for research and practice. These include testing what prompts are effective in instigating assessment of obesity (without upsetting patients), examining the relative impact of earlier intervention with overweight (but not yet obese) patients, and examining the cost-effectiveness and efficacy of different advice and referral options. These steps may help to increase provision of weight management conversations in primary care, enhance the preventative input these services have, and overcome some of the reported barriers.

## Data Availability

The datasets used and/or analysed during this study are available from the corresponding author, or the Cancer Policy Research Centre at Cancer Research UK, on reasonable request.

## Abbreviations

AOR: Adjusted Odds Ratio
BMI: Body Mass Index
CCG: Clinical Commissioning Group
CI: Confidence Intervals
GP: General Practitioner
n.s.: No significance (i.e. *p <* 0.05)
NICE: National Institute for Clinical Excellence
PN: Practice Nurse
UK: United Kingdom
